# How to continue lipid-lowering therapy in patients with coronary heart disease and severe liver dysfunction? A case report of plaque regression by a combination of Chinese and Western Medicine

**DOI:** 10.1097/MD.0000000000017664

**Published:** 2019-10-25

**Authors:** Dong Yan, Xiang-Ru Xu, Bo-Wen Yue, Li-Li Zhao, Shi-Hai Yan, Yu-Liang Qian

**Affiliations:** a Department of Cardiology, Jiangsu Province Hospital of Chinese Medicine; b Affiliated Hospital of Nanjing University of Chinese Medicine; c Department of Medicine, Jiangsu Province Hospital of Chinese Medicine; d Department of Pharmacology, Jiangsu Province Hospital of Chinese Medicine, Nanjing, China.

**Keywords:** atherosclerosis, combination of Chinese and Western medicine, coronary heart disease, plaque regression, severe liver dysfunction

## Abstract

**Rationale:**

Atherosclerotic cardiovascular disease (ASCVD), including coronary heart disease (CHD), atherosclerotic stroke and peripheral vascular disease, has become the most deadly chronic noncommunicable disease throughout the world in recent decades, while plaque regression could reduce the occurrence of ASCVD. Traditional Chinese Medicine (TCM) has been widely used for prevention and treatment of these diseases. In the perspective of TCM, phlegm and blood stasis are considered to be leading pathogenesis for CHD. Hence, activating blood circulation and dissipating phlegm, which is of great benefit to regress plaque, have been regarded as general principles in treatment.

**Patient Concerns:**

A 36-year-old man presented with a 3-month history of intermittent exertional chest pain. Coronary angiography revealed 60% stenosis of the proximal left anterior descending coronary artery. Liver function showed: alanine transaminase (ALT):627U/L, aspartate transaminase (AST):243U/L.

**Diagnoses:**

CHD and hepatitis B with severe liver dysfunction.

**Interventions:**

The patient should have been treated with high-intensity statin therapy. Actually, due to severe liver dysfunction, Huazhirougan granule instead of statins was administered. In addition, he was treated with TCM according to syndrome differentiation for two and a half years.

**Outcomes:**

The chest pain disappeared and other symptoms alleviated as well after treatment. Coronary computed tomographic angiography revealed no stenosis in the proximal left anterior descending coronary artery. ALT and AST level returned to normal (ALT:45U/L,AST:24U/L).

**Lessons:**

For patients with CHD and severe hepatic dysfunction, antilipidemic drugs such as statins are not recommended. This case suggested that TCM might fill a gap in lipid-lowering therapy. Thus, we could see that statins were not the only drug for plaque regression and the effect of TCM in treating coronary artery disease cannot be ignored.

## 1 Introduction

Recent decades, ASCVD (CHD, atherosclerotic stroke and peripheral vascular disease), has become the most deadly chronic noncommunicable disease globally.^[1]^ Atherosclerosis is a systemic disease, which is considered as pathological basis of ASCVD. Modern studies indicated that it is closely related to lipid infiltration, inflammation, oxidative stress, endothelial dysfunction, smooth muscle cell proliferation, immunity, gene, and heredity. Furthermore, hypertension, diabetes, hyperlipidemia, obesity, diet, and mental stress may play a role in the occurrence of disease. The ultimate outcome of coronary atherosclerosis is the formation of thrombosis, which might result in vascular stenosis or even plaque rupture leading to acute coronary syndrome. So, plaque regression could prevent the occurrence of ASCVD. And it referred to the reduction of both lipid nucleoli and plaque volume. Meanwhile, proliferation and phenotypic transformation of smooth muscle cells was suppressed, vascular endothelial cells were repaired and the arterial wall returned to normal. Many clinical trials demonstrated that decreasing Low-Density Lipoprotein-Cholesterol (LDL-C) and increasing High-Density Lipoprotein-Cholesterol(HDL-C) can promote cholesterol reverse transport and achieve plaque regression.^[2]^ Other studies suggested that monocytes and dendritic cells can transport plaque out of the intima via vascular-associated lymphoid tissue. Therefore, macrophage migration is closely related to plaque regression.^[3]^

CHD was called as “Xiong Bi” in TCM. It believed that blood stasis and phlegm were the pathological products of disease. Hyperlipidemia, lipid deposition and atherosclerosis resulted from phlegm, and vascular wall injury and thrombus is the expression of blood stasis. Phlegm and blood stasis obstructing vessels has become leading pathogenesis of CHD. A study found that compared with that balanced constitution, there were 355 differentially expressed genes in phlegm-dampness constitution. Consequently, patients with phlegm-dampness are most likely to suffer from atherosclerosis.^[4]^ Inflammation, endothelial injury, vascular proliferation, stenosis, and atherosclerotic plaque is easier to occur among CHD patients with the syndrome of the intermingling of phlegm and static blood.^[5]^ Therefore, activating blood circulation and dissipating phlegm became general principles of treatment. In recent years, TCM has been widely used in the prevention and treatment of CHD in China. However, there are no case reports about plaque regression by taking TCM. In this report, we present a successful case of plaque regression with severe liver dysfunction by a combination of Chinese and Western Medicine treatment on a 36-year-old man.

## 2 Case presentation

### 2.1 TCM to treat CHD

**Figure 1:**
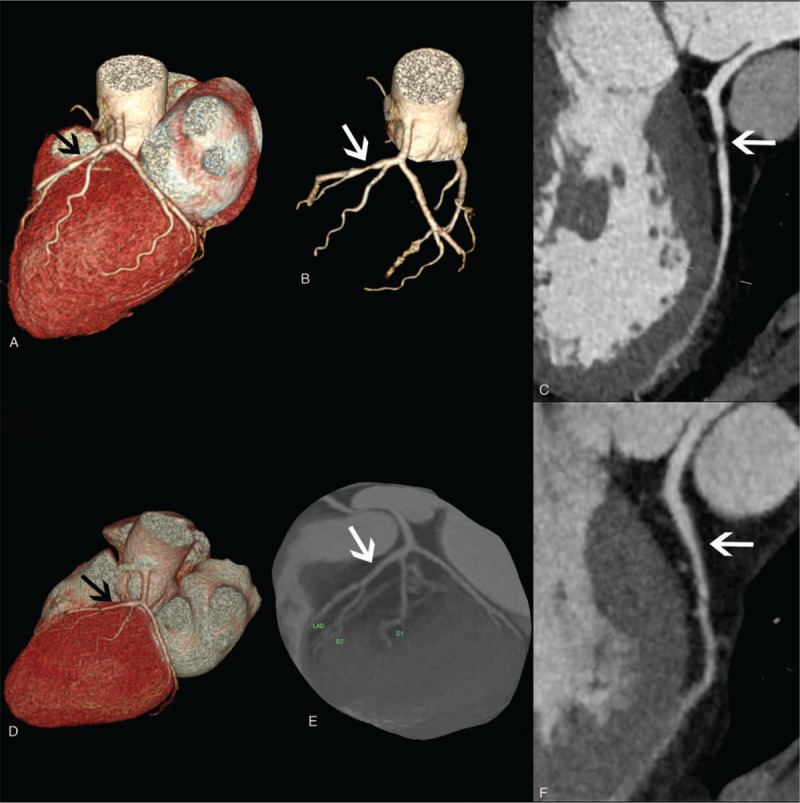
(A, B, C) Coronary angiography revealed 60% stenosis of the proximal left anterior descending coronary artery (arrows). (D, E, F) Coronary computed tomographic angiography revealed no stenosis in the proximal left anterior descending coronary artery (arrows).

A 36-year-old man was admitted to the local hospital with a 2-month history of intermittent chest pain on December 29, 2015. Besides, he also had a history of hypertension and fatty liver. Coronary angiography revealed 60% stenosis of the proximal left anterior descending coronary artery, the right coronary artery originating from the left coronary sinus (Fig. 1 A–C). He was diagnosed as CHD and treated with antiplatelet, lipid regulation and antihypertensive therapy without percutaneous coronary intervention. The symptoms were temporarily relieved. However, one and a half months later, chest pain attacked again due to exertion so that the patient went to our outpatient department for treatment. He complained shortness of breath, sleep difficulties and restless, soreness of waist and afraid of cold. Nevertheless, the patient had stayed up late for a long time. According to the physical examination, blood pressure was 135/80 mm Hg and the body-mass index (the weight in kilograms divided by the square of the height in meters) was 30.12. The patient showed a dark complexion, a pale-puffy tongue, a red tongue tip with cracks, a thin and yellow tongue coating, also thready and stringy pulse. The prescription was as follows: isosorbide mononitrate 40 mg/d, aspirin 100 mg/d, bisoprolol 2.5 mg/d, candesartan 8 mg 2 times/d, Xuezhikang capsule 0.6 g 2 times/d; Xinyuan capsule 1.2 g 3 times/d, Yixinshu capsule 1.6 g 3 times/d, Yanghuo Sanqi capsule 1.2 g 2 times/d.

Table 1 showed compositions associated with mechanisms of Yixinshu capsule (approval number: Z52020038), Xinyuan capsule (approval number: Z10970090), Yanghuo Sanqi capsule (approval number: Z20090102), Xuezhikang capsule (approval number: Z10950029).

**Table 1:**
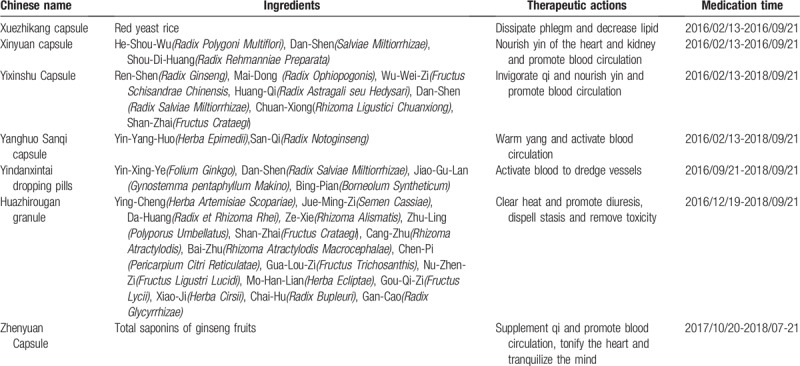
Introduction of the Chinese patent medicine taken by the patient in the past two and a half years.

TCM holds the opinion that Qi, constituting the human body and supporting life activities, is the most basic substance. It includes oxygen inhaled through 2 lungs, nutrients absorbed by the gastrointestinal tract, Adenosine Triphosphate (ATP) generated by cells. Because this man suffered from this state for a long time and lacking of sleep resulted in cardiometabolic disorders and ATP reduction. These contributed to Qi deficiency syndrome, which manifested as a pale and puffy tongue. Besides, insufficient ATP and metabolic disorder reduced the body nutrients and weakened repair function of cells. And then a series of Yin deficiency symptoms, such as a red tongue tip with cracks and thready-stringy pulse arised from these. Based on this, the cells began to degrade and the function of the cardiomyocytes was impaired. Decreasing myocardial contractility, slower blood flow, circulatory disturbances and blood stasis brought about chest pain and then dark complexion followed. Once the blood stasis formed, ischemia and hypoxia of tissues would be aggravated, ATP reduction, which made severe Yang deficiency exhibiting as soreness of waist and afraid of cold. On the other hand, body constitution of the patient belonged to phlegm-dampness because of obesity and fatty liver.^[6]^

### 2.2 How to continue lowering blood lipid after liver damage?

Seven months after taking the medicine, the patient complained that chest pain disappeared, blood pressure was normal, sleep quality was improved, red tongue tip with cracks disappeared and the pulse normalised. But physical examination showed liver dysfunction. Statins and angiotensin receptor blockers may cause an increase of liver enzymes but different drugs had different effects on liver function. Thus, xuezhikang or candesartan may not be suitable for this patient. Firstly, we thought about that replacing with another statin and antihypertensive drug, so he was given amlodipine and atorvastatin calcium tablets with a daily dosage of 5 mg/10 mg, respectively to control blood pressure and regulate lipid simultaneously. Meanwhile, antiviral drugs and hepatoprotective drugs were administered. Considering the possible progression of coronary artery stenosis after reducing statins dosage, Xinyuan capsule was changed with Yindanxintai dropping pills (12 pills three times daily; approval number: Z20025687) for enhancing the effect of promoting blood circulation and removing blood stasis.

However, his liver function still did not return to normal level after changing the treatment plan. More than a month later, the patient was diagnosed as hepatitis B (HBsAg+, HBeAb+, HBcAb+) in the local hospital. Liver function showed: alanine transaminase (ALT):627U/L, aspartate transaminase (AST):243U/L. According to 2016 ESC/EAS Guidelines for the Management of Dyslipidaemias,^[7]^ when ALT > 3 times of the upper limit of normal, statins should be discontinued during hepatic dysfunction period and be reused when transaminase returned to normal. Therefore, amlodipine and atorvastatin calcium tablets were stopped. As the reason of liver dysfunction is clearly associated with hepatitis B, candesartan could be given.

The question is that how to reduce blood lipids during the withdrawal of antilipidemic drugs? A clinical trial in China reported that Huazhirougan granule (approval number: Z20090077) could reduce ALT, AST, total cholesterol (TC) and triglyceride(TG) levels significantly and protect liver function with high safety.^[8]^ Consequently, prescriptions were changed as follows: isosorbide mononitrate sustained 40 mg/d, aspirin 100 mg/d, bisoprolol 2.5 mg/d, Yixinshu capsules 1.6 g/tid, Yanghuosanqi capsules 1.2 g/bid, Yindanxintai dropping pills 12 pills/tid, Huazhirougan granule 8 g/bid, entecavir 0.5 mg/d and silymarin 70 mg/tid.

Seven months later, the chest pain did not occur for a long time. ALT, AST returned to normal (ALT:45U/L, AST:24U/L). In the absence of regular statins, no plaque regression was ever reported within 15 months, so coronary angiography was not performed. Considering carotid artery plaque could predict the presence of coronary plaque to a certain extent,^[9]^ the carotid artery examination was made for the patient. The carotid ultrasound showed no plaque formation, so it can be inferred that coronary plaque may not progress. He restarted amlodipine atorvastatin (5 mg/10 mg) since the liver function returned to normal. But the electrocardiogram displayed sinus bradycardia. Besides, the pale-puffy and teeth-printed tongue along with night sleepless indicated that symptoms of Qi deficiency were still obvious. Thus, we chose Zhenyuan capsule (50 mg 3 times/day; approval number: Z22026091) which contained ginsenoside to enhance the patient's myocardial contractility and heart rate. This drug can also help improve sleep. A study has revealed that Zhenyuan capsule is effective and safe for CHD with abnormal lipid metabolism.^[10]^

### 2.3 Plaque regression

The patient had taken medicine for about two and a half years. Table 1 showed the Chinese patent medicine that was taken by the patient over the past years. On July 21, 2018, coronary computed tomographic angiography (CTA) reexamination was performed on the patient. The results showed that the left anterior descending coronary artery wall was smooth. There is no thickening, no calcification and no stenosis of the artery. The right coronary artery originated from the left coronary sinus. It indicated that the coronary plaque was regressed. (Fig. 1 D–F) The patient was suggested to take these medications for another 2 months. Then, he stopped taking all drugs completely. So far, the condition of patient was stable and no cardiovascular events or adverse reactions occurred.

## 3 Discussion

According to Chinese new published “guidelines for the diagnosis and treatment of stable CHD”,^[11]^ patients should be advised to control diet, lifestyle and risk factors first. Second, nitrates, beta-receptor blockers, calcium channel blockers are given to improve symptoms while aspirin, statins, angiotensin receptor blocker are given to improve prognosis.

So many clinical trials indicated that high-intensity statin therapy can regress atherosclerotic plaque and reduce ASCVD events with decreasing LDL-C. Current evidence suggested that stains, such as atorvastatin and rosuvastatin can regress plaque, so sid statins combined with ezetimibe.^[12]^ The ASTEROID trial,^[13]^ IBIS 4^[14]^ and SATURN study^[15]^ in America-European population reported that high-intensity statin therapy(40 mg/day rosuvastatin, 80 mg/day atorvastatin) could regress atherosclerosis. The COSMOS investigation^[16]^ (average 16.9 mg/day rosuvastatin) and ARTMAP trail^[17]^ (10 mg/day rosuvastatin, 20 mg/day atorvastatin) in Asian population stated that moderate or low-intensity statin can reduce LDL-C, increase HDL-C and regress plaque. The CHILLAS study in China demonstrated that intensive statin therapy did not exhibit significant clinical effectiveness compared to moderate statin therapy.^[18]^ Due to high sensitivity of Asians to statins, the recommended dosage of statin therapy in Chinese expert consensus is 10 mg/day rosuvastatin or 20 mg/day atorvastatin.^[19]^

In this case, we first chose Xuezhikang, composed of amorphous lovastatin with high safety, could decrease LDL-C, small dense LDL, apolipoprotein B, Lipoprotein(a) and increase HDL-C, apolipoprotein A-1.^[20]^ It can also protect endothelial function through mechanisms of antiinflammatory and lipid-lowering.^[21]^ The patient was actually treated with Xuezhikang (containing 10 mg lovastatin) for 7 months. Atorvastatin(10 mg) substitute for it due to hepatic dysfunction. However, the patient was finally diagnosed as hepatitis B with a severe elevation of aminotransferase. Statins was forbidden for active liver disease. Ezemebuk and Xuezhikang also showed detrimental effects on liver function. Therefore, Huazhirougan granule exhibited significant advantage on reducing lipid with high safety. When liver function returned to normal after 7 months, he re-started a low-intensity of 10 mg atorvastatin combined with Huazhirougan granule for another 14 months. Not only the symptoms were relieved but also the coronary plaque was regressed. This case suggested that TCM might fill the gap in lipid-lowering therapy for CHD patients with severe hepatic dysfunction when antilipidemic drugs, such as statins was not advised. On the other hand, statins may not be the only drug for plaque regression. The effect of TCM in treating coronary artery disease should not be ignored. There was also a special point in the treatment of this patient. Aspirin was an antipyretic, analgesic and anti-inflammatory drug, which could resist platelet aggregation and widely used for treating coronary atherosclerosis. However, TCM believed that it belonged to cold natural medicine for clearing away heat, promoting blood circulation for removing blood stasis. This patient was identified as Yang deficiency and the risk of gastrointestinal bleeding may increase if he continued taking aspirin. Thus, Yanghuosanqi capsule was chosen to avoid side effects rather than aspirin. No adverse reactions occurred and the therapeutic effect was satisfactory. Did this result mean that western medicine could be given according to syndrome differentiation of herbalist doctor?

Among these Chinese patent medicine, Fructus Crataegi (Shan-Zhai), Rhizoma Alismatis (Ze-Xie), Radix et Rhizoma Rhei (Da-Huang), Semen Cassiae (Jue-Ming-Zi), Red yeast rice, Radix Polygoni Multiflori (He-Shou-Wu), Radix Salviae Miltiorrhizae (dan shen), Rhizoma Ligustici Chuanxiong (Chuan-Xiong), and Radix Astragali seu Hedysari (Huang-Qi) exhibited apparent lipid-lowering effects.^[22]^ Besides, Danshensu Bingpian Zhi revealed an antiatherosclerotic effect on the human body through inhibiting inflammation, macrophage migration, leukocyte adhesion, and formation of foam cells.^[23]^ Ginsenoside Rb1 could reduce the accumulation of lipid in macrophage foam cells and enhance the stability of atherosclerotic plaque by inducting macrophage autophagy.^[24]^ Even so, mechanism of plaque regression is difficult to be explained clearly. There is more than 2000 years history of TCM, which are various monomers with complex mechanisms, multi-channels and multi-targets in Chinese medicine. Modern studies are inclined to extract pharmaceutical ingredients from Chinese herds. Actually, this method is worth debating. Once Chinese herds enter the gastrointestinal tract, intestinal microbes can generate new components from them. After metabolism of human body, even new ingredients were generated by different kinds of herds, which could play a new therapeutic effect.^[25]^ Relying on that to prevent or treat disease is one of the major characteristics of TCM. Generally speaking, the efficacy of TCM is complex. Chinese medicine can play the best role in treatment under the guidance of TCM theory. This is also the key point for the successful case.

## 4 Conclusion

As we all known, intervention on the early stage of plaque formation (soft plaque) can regress and eliminate plaques completely. While lipid nuclei and fibrous caps shaped on the later stage of plaque formation (mature plaque), which is difficult to regress, for there are more foam components and fewer cells in the plaque. It is common clinically. The shortcomings in this report is that neither coronary angiography nor CTA could monitor composition of plaque. We have no idea which type of plaque has been regressed. At present, the mechanism of plaque regression is still complicated for us. It is still needed to be solved that which components in plaques are easy to be regressed, which pathway can make plaques regress, and whether there is any change in the structure of plaques after plaque regression. In addition, we should take full advantage of TCM with its multi-directional and multi-target regulatory effects. It is meaningful to research the mechanism of TCM and find new targets of drugs in regressing plaque so that we can have more choice in the treatment of atherosclerosis.

## Author contributions

**Conceptualization:** Yue Bowen, Zhao Lili.

**Data curation:** Xu Xiangru, Yue Bowen, Zhao Lili.

**Formal analysis:** Yan Dong, Xu Xiangru.

**Investigation:** Xu Xiangru.

**Methodology:** Yuliang Qian.

**Project administration:** Yan Shihai, Yuliang Qian.

**Resources:** Yan Dong.

**Visualization:** Yan Shihai.

**Writing – original draft:** Xu Xiangru.

**Writing – review & editing:** Yan Dong.

yuliang qian orcid: 0000-0001-8337-1277.

## References

[1] Grundy SM Arai H Barter P An international atherosclerosis society position paper: global recommendations for the management of dyslipidemia--full report. J Clin Lipidol 2014;8:29 60. 24528685 10.1016/j.jacl.2013.12.005

[2] Feig JE Rong JX Shamir R HDL promotes rapid atherosclerosis regression in mice and alters inflammatory properties of plaque monocyte-derived cells. Proc Natl Acad Sci U S A 2011;108:7166 71. doi: 10.1073/pnas.1016086108[published Online First: Epub Date]. 21482781 10.1073/pnas.1016086108 PMC3084076

[3] Llodra J Angeli V Liu J Emigration of monocyte-derived cells from atherosclerotic lesions characterizes regressive, but not progressive, plaques. Proc Natl Acad Sci U S A 2004;101:11779 84. doi: 10.1073/pnas.0403259101[published Online First: Epub Date]. 15280540 10.1073/pnas.0403259101 PMC511052

[4] Li L Feng J Yao H Gene expression signatures for phlegm-dampness constitution of Chinese medicine. Sci China Life Sci 2017;60:105 7. doi: 10.1007/s11427-016-0212-9[published Online First: Epub Date]. 27928700 10.1007/s11427-016-0212-9

[5] Liu YJ Hu JQ Jiang LJ Research status of molecular biological mechanism of phlegm-blood stasis interaction in coronary heart disease. World Sci Technol Mod Tradit Chin Med 2016;18:791 9.

[6] Tang S Ma X Song T Study progress on reversal of atherosclerosis plaque by traditional Chinese medicine and its preparation. Shandong J Tradit Chin Med 2014;33:322 5.

[7] Catapano AL Graham I De Backer G 2016 ESC/EAS guidelines for the management of dyslipidaemias. Eur Heart J 2016;37:2999 3058. doi: 10.1093/eurheartj/ehw272[published Online First: Epub Date]. 27567407 10.1093/eurheartj/ehw272

[8] Yang S Guo Y Li T Huazhi rougan granule in treating damp-heat accumulation type non-alcoholic fatty liver disease. Chin J Exp Tradit Med Formulae 2015;21:157 60.

[9] Shah PK Can carotid plaque predict coronary plaque? JACC Cardiovasc Imaging 2013;6:1168 71. doi: 10.1016/j.jcmg.2013.09.005[published Online First: Epub Date]. 24229769 10.1016/j.jcmg.2013.09.005

[10] Qiao Y Zhang J Liu Y Efficacy and safety of zhenyuan capsule for coronary heart disease with abnormal glucose and lipid metabolism: study protocol for a randomized, double-blind, parallel-controlled, multicenter clinical trial. Evid Based Complement Alternat Med 2018;2018:1716430 doi: 10.1155/2018/1716430[published Online First: Epub Date]. 29853944 10.1155/2018/1716430 PMC5944192

[11] Chinese Society of Cardiology, Interventional Cardiology Group; Chinese Society of Cardiology, Atherosclerosis and Coronary Heart Disease Group; Committee of Chinese Doctor Association, Cardiology of Thrombosis Prevention and Treatment, et al. Guidelines for the diagnosis and treatment of stable Coronary artery Disease. Chin J Cardiol 2018;46:680 94.

[12] Tsujita K Sugiyama S Sumida H Impact of dual lipid-lowering strategy with ezetimibe and atorvastatin on coronary plaque regression in patients with percutaneous coronary intervention: the multicenter randomized controlled PRECISE-IVUS trial. J Am Coll Cardiol 2015;66:495 507. doi: 10.1016/j.jacc.2015.05.065[published Online First: Epub Date]. 26227186 10.1016/j.jacc.2015.05.065

[13] Nissen SE Nicholls SJ Sipahi I Effect of very high-intensity statin therapy on regression of coronary atherosclerosis: the ASTEROID trial. JAMA 2006;295:1556 65. doi: 10.1001/jama.295.13.jpc60002[published Online First: Epub Date]. 16533939 10.1001/jama.295.13.jpc60002

[14] Raber L Taniwaki M Zaugg S Effect of high-intensity statin therapy on atherosclerosis in non-infarct-related coronary arteries (IBIS-4): a serial intravascular ultrasonography study. Eur Heart J 2015;36:490 500. doi: 10.1093/eurheartj/ehu373[published Online First: Epub Date]. 25182248 10.1093/eurheartj/ehu373

[15] Nicholls SJ Ballantyne CM Barter PJ Effect of two intensive statin regimens on progression of coronary disease. New Engl J Med 2011;365:2078 87. doi: 10.1056/NEJMoa1110874[published Online First: Epub Date]. 22085316 10.1056/NEJMoa1110874

[16] Takayama T Hiro T Yamagishi M Effect of rosuvastatin on coronary atheroma in stable coronary artery disease: multicenter coronary atherosclerosis study measuring effects of rosuvastatin using intravascular ultrasound in Japanese subjects (COSMOS). Circ J 2009;73:2110 7. 19801853 10.1253/circj.cj-09-0358

[17] Lee CW Kang SJ Ahn JM Comparison of effects of atorvastatin (20 mg) versus rosuvastatin (10 mg) therapy on mild coronary atherosclerotic plaques (from the ARTMAP trial). Am J Cardiol 2012;109:1700 4. doi: 10.1016/j.amjcard.2012.01.399[published Online First: Epub Date]. 22440123 10.1016/j.amjcard.2012.01.399

[18] Zhao SP Yu BL Peng DQ The effect of moderate-dose versus double-dose statins on patients with acute coronary syndrome in China: results of the CHILLAS trial. Atherosclerosis 2014;233:707 12. doi: 10.1016/j.atherosclerosis.2013.12.003[published Online First: Epub Date]. 24603217 10.1016/j.atherosclerosis.2013.12.003

[19] Liao Y Cheng X Huang K Expert consensus on statin-induced plaque regression in patients with atherosclerotic cardiovascular disease. J Clin Cardiol 2015;31:1 5.

[20] Committee of Cardio-Cerebro-Vascular Diseases of Gerontological Society of China, Working Group of Chinese Expert Consensus on the Use of Xuezhikang. Chinese expert consensus on the use of Xuezhikang (2017 revised edition). Zhonghua Nei Ke Za Zhi 2018;57:97 100. doi: 10.3760/cma.j.issn.0578-1426.2018.02.003[published Online First: Epub Date]. 29397593 10.3760/cma.j.issn.0578-1426.2018.02.003

[21] Zhao SP Liu L Cheng YC Xuezhikang, an extract of cholestin, protects endothelial function through antiinflammatory and lipid-lowering mechanisms in patients with coronary heart disease. Circulation 2004;110:915 20. doi: 10.1161/01.Cir.0000139985.81163.Ce[published Online First: Epub Date]. 15313947 10.1161/01.CIR.0000139985.81163.CE

[22] Wang C Niimi M Watanabe T Treatment of atherosclerosis by traditional Chinese medicine: questions and quandaries. Atherosclerosis 2018;277:136 44. doi: 10.1016/j.atherosclerosis.2018.08.039[published Online First: Epub Date]. 30212682 10.1016/j.atherosclerosis.2018.08.039

[23] Wang J Xu P Xie X DBZ (Danshensu Bingpian Zhi), a novel natural compound derivative, attenuates atherosclerosis in apolipoprotein e-deficient mice. J Am Heart Assoc 2017;6: doi: 10.1161/jaha.117.006297[published Online First: Epub Date]. 10.1161/JAHA.117.006297 PMC5721843 28971954

[24] Qiao L Zhang X Liu M Ginsenoside Rb1 enhances atherosclerotic plaque stability by improving autophagy and lipid metabolism in macrophage foam cells. Front Pharmacol 2017;8:727 doi: 10.3389/fphar.2017.00727[published Online First: Epub Date]. 29114222 10.3389/fphar.2017.00727 PMC5660703

[25] Zhu SN Conversion taking effect: complex efficacy mechanism of traditional Chinese medicine. J Shandong Univ Tradit Chin Med 2008;32:179 81.

